# Anti-Aging and Neuroprotective Properties of *Grifola frondosa* and *Hericium erinaceus* Extracts

**DOI:** 10.3390/nu14204368

**Published:** 2022-10-18

**Authors:** Farida Tripodi, Ermelinda Falletta, Manuela Leri, Cristina Angeloni, Daniela Beghelli, Laura Giusti, Riccardo Milanesi, Belém Sampaio-Marques, Paula Ludovico, Lorenzo Goppa, Paola Rossi, Elena Savino, Monica Bucciantini, Paola Coccetti

**Affiliations:** 1 Department of Biotechnology and Biosciences, University of Milano-Bicocca, 20126 Milano, Italy; 2 Department of Chemistry, University of Milano, 20133 Milano, Italy; 3 Department of Experimental and Clinical Biomedical Sciences, University of Firenze, 50134 Firenze, Italy; 4 Department for Life Quality Studies, Alma Mater Studiorum, University of Bologna, 47921 Rimini, Italy; 5 School of Biosciences and Veterinary Medicine, University of Camerino, 62032 Camerino (MC), Italy; 6 School of Pharmacy, University of Camerino, 62032 Camerino (MC), Italy; 7 Life and Health Sciences Research Institute (ICVS), School of Medicine, University of Minho, 4710-057 Braga, Portugal; 8 ICVS/3B’s-PT Government Associate Laboratory, 4806-909 Braga/Guimarães, Portugal; 9 Department of Earth and Environmental Sciences (DSTA), University of Pavia, 27100 Pavia, Italy; 10 Department of Biology and Biotechnology “L. Spallanzani” (DBB), University of Pavia, 27100 Pavia, Italy

**Keywords:** *Saccharomyces cerevisiae*, α-synuclein, *Grifola frondosa*, *Hericium erinaceus*, medicinal mushrooms, Parkinson’s disease (PD), Drosophila melanogaster

## Abstract

Nutrition has relevant consequences for human health and increasing pieces of evidence indicate that medicinal mushrooms have several beneficial effects. One of the main issues in Western countries is represented by the challenges of aging and age-related diseases, such as neurodegenerative disorders. Among these, Parkinson’s disease (PD) affects 10 million people worldwide and is associated with α-synuclein misfolding, also found in other pathologies collectively called synucleinopathies. Here, we show that aqueous extracts of two edible mushrooms, *Grifola frondosa* and *Hericium erinaceus*, represent a valuable source of β-glucans and exert anti-aging effects in yeast. Their beneficial effects are mediated through the inhibition of the Ras/PKA pathway, with increased expression of heat shock proteins, along with a consistent increase of both mean and maximal lifespans. These fungal extracts also reduce the toxicity of α-synuclein heterologously expressed in yeast cells, resulting in reduced ROS levels, lower α-synuclein membrane localization, and protein aggregation. The neuroprotective activity of *G. frondosa* extract was also confirmed in a PD model of *Drosophila melanogaster*. Taken together, our data suggest the use of *G. frondosa* and *H. erinaceus* as functional food to prevent aging and age-related disorders, further supporting the neuro-healthy properties of these medicinal mushroom extracts.

## 1. Introduction

The budding yeast *Saccharomyces cerevisiae* is one of the most used model organisms to study molecular mechanisms of several cellular processes, from cell cycle to metabolism and signaling, due to the high evolutionary conservation of these processes with higher eukaryotes and due to its simple genetic manipulation. In particular, the yeast chronological life span (CLS) assay has been successfully used to model the aging of post-mitotic cells of higher organisms [1,2]. Many different models using yeast strains overexpressing human genes associated with diseases like Alzheimer’s disease (AD), Parkinson’s disease (PD) or Hungtington’s disease (HD) have also been exploited in the last decades. Among them, one of the most studied yeast models consists on the expression of α-synuclein (α-syn) [3,4,5,6], a presynaptic protein whose alteration is associated with synucleinopathies, such as PD [7,8,9].

Another simple but powerful model for studying aging and neurodegenerative diseases is the invertebrate *Drosophila melanogaster*, whose common name is fruit fly. *D. melanogaster* is characterized by a well-defined nervous system and around 75% of the genes responsible for human diseases are evolutionarily conserved in *Drosophila* [10]. Transgenic flies expressing human wild-type (WT) α-syn are a PD model that has been experimentally shown to mimic several aspects of this neurodegenerative disease [11].

Nowadays, there is increasing attention on nutrition as a means of preventing some human pathologies, since natural compounds can be used in everyday diet with few side effects. Mushrooms’ nutritional value is evidenced by their low caloric value and fat composition, whereas they are rich in proteins, fibers, carbohydrates, vitamins, and minerals. Medicinal mushrooms have been used since the Neolithic age by humans for their beneficial effects on health and about 270 species are known for their preventive or therapeutic properties [12,13]. Because of their potential effects on human health, two of the most studied species are *Grifola frondosa* (Dicks.) Gray and *Hericium erinaceus* (Bull.) Pers [14].

*Grifola frondosa*, also referred to as Maitake, is an edible wood-decay mushroom that grows mainly on oaks or chestnuts, used as a health food for centuries in China and Japan. The medicinal value of this mushroom concerns its high content on polysaccharides, all above β-glucans, that are responsible for its immunostimulating, anti-tumoral, anti-diabetic, anti-allergic, anti-oxidant, anti-aging, and neuroprotective effects [15,16].

*Hericium erinaceus*, also known as Yamabushitake or lion’s mane mushroom, is a culinary medicinal mushroom that grows mainly on oaks or holm-oaks, with interesting therapeutic activities related to the promotion of nerve and brain health [17]. Its mycelium and sporophore present an extraordinary quantity of bioactive metabolites that make this mushroom a strong anti-carcinogenic, antibiotic, anti-fatigue, cardioprotective, nephroprotective, anti-senescence, and anti-depressant species [18,19,20,21]. According to different studies, *H. erinaceus* can act by reducing the neuroinflammation associated with neurodegenerative disorders [22,23].

β-1,3-1,6 glucans from *G. frondosa* allow lifespan extension as described by the daily administration to the model organism *Nothobranchius guentheri* [24] and to *Caenorhabditis elegans* [25]. Proteo-β-glucan from *G. frondosa* has shown surprising effects on ameliorating Alzheimer’s disease-like pathologies and the associated cognitive impairment in mice through an increased Aβ plaques phagocytosis by microglia cells [26].

β-glucans from *H. erinaceus* play a key role in improving health because of their immunomodulatory, anti-tumor, and antioxidant properties by scavenging reactive oxygen species. All those effects could reduce the risk of diseases, including neurodegenerative diseases [27,28]. β-glucans belong to the group of prebiotics with an important effect on the ecological fitness of our gut microbiome, thus exerting a neuroprotective role through the microbiota–gut–brain axis [29].

L-ergothioneine (ET) is a thiol/thione derivative from histidine, found in large quantities in mushrooms [30], and called the “longevity vitamin” for its health-promoting effect [31,32,33]. From a nutritional point of view, ET is essential for humans because it is only acquired from diet, and it is easily absorbed in the gut of humans via the specific organic cation transporter (OCTN1), thus reaching high levels in the body [34]. ET displays antioxidant activity by scavenging ROS (reactive oxygen species) and by activating antioxidant enzymes, and it is described as a neuroprotective agent against protein aggregation in PD [35,36]. It has been reported that a deficiency in ET blood levels increases the risk of several neurodegenerative disorders including PD, mild cognitive impairment (MCI), and frailty in aging [37]. The cause–effect relationship between this ET decline and the risk of neurodegenerative diseases remains to be established.

Herein, we explored the anti-aging and protective effects of *G. frondosa* and *H. erinaceus* aqueous extracts on the model organism *S. cerevisiae.* The chemical composition of both extracts was analyzed, with a focus on polysaccharides, free amino acids, and ET content. Furthermore, the neuroprotective activity of *G. frondosa* was also investigated in a transgenic model of *Drosophila melanogaster* expressing human α-synuclein. These mushrooms were found to exert anti-aging effects and reduce the toxicity of α-synuclein in the yeast model, showing also neuroprotective activity in a PD model of *Drosophila melanogaster*. Thus, *G. frondosa* and *H. erinaceus* extracts have great potential to prevent aging and age-related disorders.

## 2. Materials and Methods

### 2.1. Chemical Reagents

All chemicals of analytical grade were purchased from Sigma-Aldrich (Milano, Italy). For both HPLC/MS analyses, HPLC-grade acetonitrile and water were purchased from VWR Chemicals (Milano, Italy) and Carlo Erba (Milano, Italy), respectively.

### 2.2. Extract Preparation

The dried fruiting bodies of *G. frondosa* and *H. erinaceus* were provided by Mycology Laboratory (DSTA) of Pavia University (Italy) and then reduced in a fine powder. Then, 2 g of dry powder was suspended in 60 mL of ultrapure MilliQ water. Then, extracts were prepared through a magnetic stirrer at 500 rpm for 60 min at 100 °C. The extraction was repeated three times. Then, it was centrifuged at 5000 g for 30 min, and the supernatant was recovered and freeze-dried.

### 2.3. Thermogravimetric Analysis (TGA)

TGA analyses were performed in nitrogen (10 °C/min, temperature range 30–700 °C) with a TGA 4000 Perkin Elmer (Milano, Italy) instrument equipped with a 70 µL alumina crucible.

### 2.4. Attenuated Total Reflectance (ATR) Fourier Transform Infrared (FT-IR) Spectroscopy

The ATR-FTIR spectra of the samples were recorded using a Bruker Vertex 70 spectrophotometer (Bruker, Billerica, MA, USA) equipped with the Harrick MVP2 ATR cell (resolution 4 cm^−1^).

### 2.5. Free Amino Acids and Ergosterol Identification

Both the samples were subjected at first to a derivatization process (silanization) and then analyzed by GC/MS. More in detail, 2 mg of each sample was accurately weighed and suspended in 25 µL of 2 wt% methoxylamine hydrochloride in pyridine. The suspensions were incubated at 37 °C. After 90 min, 40 µL of MBDSTFA (N-methyl-N-ter-butyldimethylsilyl-trifluoroacetamide)+1% TBDMCS (tert-butyldimethylchlorosilane) was added and stirred at 60 °C for 30 min. Finally, after incubation at room temperature overnight, the samples were analyzed by using a ISQ™ QD Single Quadrupole GC-MS (Thermo Fisher, Milano, Italy). A VF-5ms (30 m × 0.25 mm i.d. × 0.25 µm; Agilent Technology, Milano, Italy) column was used for the chromatographic separation Injection volume: 1 µL. Oven program: 100 °C for 2 min; then 6 °C/min to 280 °C for 15 min; Run Time 42 min. Helium was used as the gas carrier. SS Inlet: Mode Splitless. Inlet temperature: 280 °C. Flow 1.0 mL/min. MS transfer line: 270 °C. Ion source: 250 °C. Ionization mode: electron impact: 70 eV. Acquisition mode: full scan. In order to compare the composition of the extracts, for each analyte identified by GC/MS, target ions were extracted by the TIC and the corresponding area was measured. Supplementary Table S1 reports the corresponding target ion used for each analyte.

### 2.6. Analysis of Monosaccharide Composition

According to the literature [38], 10 mg of each sample was dissolved in 3 mL of dried dimethylsulfoxide. Then, 40 mg of dried NaOH was added followed by 2 mL of CH_3_CI. The solution was kept under stirring for 10 min. Then, 2 mL of deionized water was added to stop the reaction. The methylated polysaccharides were extracted three times by CH_3_Cl. The organic fractions were collected together and vaporized at 40 °C. The obtained products were put in contact with 5 mL of 2 M trifluoroacetic acid at 110 °C. After 3 h, 3 mL of ethanol was added and the solution was dried by a rotary evaporator. This step was repeated three times. The partially methylated sugars in the hydrolysate reacted with 3 mL of 0.3% NaOH and 40 mg of NaBH_4_ at room temperature. After 3 h the pH was adjusted to 4 by glacial acetic acid, ethanol was added, and the solution was evaporated by the use of a rotary evaporator until dryness. This step was repeated five times. Then, 5 mL of acetic anhydride was added to the sample and the solution was heated at 100 °C for 1.5 h. Finally, partially methylated alditol acetates were extracted three times by CHCl_3_. The organic fractions were collected together, washed with deionized water, reduced in volume, and analyzed by using an ISQ™ QD Single Quadrupole GC-MS (Thermo Fisher Scientific, Milano, Italy). A VF-5ms (30 m × 0.25 mm i.d. × 0.25 µm; Agilent Technology, Milano, Italy) was used as chromatographic column. Injection volume: 1 µL. Oven program: 50 °C for 5 min; then 20 °C/min to 200 °C for 15 min; then 20 °C/min to 280 °C for 5 min; Run Time 37 min. Helium was used as the gas carrier. SS Inlet: Mode Splitless. Inlet temperature: 280 °C. Flow 1.0 mL/min. MS transfer line: 270 °C. Ion source: 280 °C. Ionization mode: electron impact: 70 eV. Acquisition mode: full scan.

### 2.7. Analysis of Ergothioneine

The identification and quantification of ergothioneine was performed by a HPLC Dionex Ultimate 3000 (Thermo Fisher Scientific, Milano, Italy) equipped with an autosampler, temperature-controlled column compartment and UV detector and interfaced with a LTQ XL (Thermo Fisher Scientific, Milano, Italy) ion trap mass spectrometer (MS) with an electrospray ionization source (ESI). Chromatographic analysis was performed by a Synergi™ Hydro-RP 80 Å (Phenomenex) column (250 × 4.6 mm, 4 µm) and with a 20 µL injection loop. Ergothioneine was separated using a gradient elution program, which consisted of water with 0.1% formic acid (Solvent A) and acetonitrile with 0.1% formic acid (Solvent B) at a steady flow rate of 0.6 mL/min. The gradient started with 99% A and 1% di B for 10 min, then in 15 min the percentage of B ramped to 70% B, whereas A dropped to 30%. The presence of ergothioneine in the compounds was also monitored at 260 nm by the UV detector. The MS interface conditions for the sample acquisition were the following: capillary temperature 275 °C, sheath gas flow rate (arb) 20, auxiliary gas flow rate (arb) 5, spray voltage 5 kV, capillary voltage 17 kV, and tube lens 75 V. MS was operated in Selected Ion Monitoring (SIM) mode (229.5–230.5 *m*/*z*) for the quantification, whereas MS^n^ analyses were carried out for the identification of the target molecule. In this last case, the parent ion was isolated with a mass width of *m*/*z* = 1 and the MS^n^ product ions spectra were produced by collision induced dissociation of the protonated molecular ion [M + H]^+^ by helium as the collision gas with a collision energy of 19%.

For LC/MS analyses, 10 mg of each sample was dissolved in 1 mL of ultrapure water and properly analyzed. An ergothioneine standard was also used as reference material. The calibration curve was constructed by injecting standard mixture solutions at four concentrations (5, 10, 25, 50 µg/mL) (Supplementary Figure S1). Each solution was analyzed in triplicate.

### 2.8. Yeast Strains and Media

The yeast strains used in this paper are listed in Supplementary Table S2. Cells were grown at 30 °C in minimal medium containing 2% glucose as a carbon source and 0.67% yeast nitrogen base without amino acids and supplemented with 50 mg/l of required amino acids and bases for which the strains were auxotrophic. The natural extracts were dissolved in the medium at a concentration of 0.2% or 0.5% and filtered through 0.22 µm filters.

### 2.9. Chronological Lifespan Experiments (CLS)

Cell cultures were grown in liquid medium until the mid-late exponential phase and then inoculated at 0.150 OD/mL in flasks containing medium in the presence or absence of the natural extracts (0.2% or 0.5% as indicated in each experiment). Survival was assessed by propidium iodide staining (PI) at the indicated time points with the Cytoflex cytofluorimeter (Beckman Coulter, Milano, Italy) and analyzed with the Cytoflex software. For some experiments, survival was also confirmed by colony-forming units (CFUs) after 2 days of incubation at 30 °C on YPD agar plates.

### 2.10. Yeast Protein Extraction, Cell Fractionation and Immunoblotting

Equal amounts of cells were collected by filtration and immediately frozen at −80 °C. Cell were resuspended in lysis buffer (50 mM Tris-HCl pH 7.5, 150 mM NaCl, 0.1% NP-40, 10% glycerol) plus 1 mM PMSF (phenylmethanesulfonylfluoride), protease inhibitor cocktail (Roche, Monza, Italy), and phosphatase inhibitor cocktail (Merk Life Science, Milano, Italy). An equal volume of acid-washed glass beads (Merk Life Science, Milano, Italy) was added, and cells were broken by 10–15 vortex/ice cycles of 1 min each. Extracts were transferred to new tubes and clarified by centrifugation. Protein concentration was determined using the Bio-Rad protein assay. The cytoplasmic-membrane fractionation experiment was conducted using the MEM-PER kit (Thermo Fisher Scientific, Milano, Italy), following the manufacturer’s instructions on yeast spheroplasts. Western blot analysis was performed using anti-Synuclein antibody (1:1000, Merk Life Science, Milano, Italy), anti-Pgk1 antibody (1:1000, Thermo Fisher Scientific, Milano, Italy, used as loading control and cytoplasmic marker) and anti-Pma1 antibody (1:1500, Abcam, Cambridge, UK, used as membrane marker). HRP-conjugated anti-rabbit and anti-mouse secondary antibodies (1:5000, Merk Life Science, Milano, Italy) were used.

### 2.11. Immunofluorescence Analysis

In situ immunofluorescence was performed on formaldehyde-fixed cells and carried using α-synuclein immunostaining (1:500, Sigma Aldrich, St. Louis, MO, USA) followed by indirect immunofluorescence using anti-rabbit Alexa Fluor488 antibody (1:500, Thermo Fisher Scientific, Milano, Italy). Digital images were taken with a Thunder Imaging System, with an oil 100X oil objective (Leica, Milano, Italy).

### 2.12. Heat Shock Assay

Yeast cells were grown in minimal medium until the mid-late exponential phase and then inoculated at 0.150 OD/mL in flasks containing medium in the presence or absence of 0.5% fungal extracts. After 24 h treatment, cells were diluted at 2.5 × 10^6^ cells/mL and 0.5 mL were treated at 51 °C in a water bath for 0, 5, 10, 15, 20, 25, 30 min (heat shock). Then, cells were diluted 1:10, spotted on YPD plates, and grown at 30 °C for 2 days.

### 2.13. Analysis of Reactive Oxygen Species (ROS) Levels

The analysis of cellular superoxide was performed by DHE (dihydroethidium) staining. Cells were collected after 24 h treatment with 0.2% fungal extract and 0.2 OD were resuspended in PBS and stained with 5 μg/mL for 10 min. FACS analyses were performed with a Cytoflex cytofluorimeter (Beckman Coulter, Milano, Italy) and analyzed with the Cytoflex software.

### 2.14. Analysis of Aggresomes

Analysis of intracellular protein aggresomes was performed with the PROTEOSTAT^®^ Aggresome detection kit (ENZO Life Sciences, Euroclone, Milano, Italy). Cells were collected after 24 h treatment with 0.2% fungal extract and 0.2 OD were resuspended in assay buffer 1x and stained with PROTEOSTAT^®^Aggresome detection reagent at a dilution 1:15,000. FACS analyses were performed with a Cytoflex cytofluorimeter (Beckman Coulter, Milano, Italy) and analyzed with the Cytoflex software.

### 2.15. RNA Extraction and qRT-PCR

RNA extraction was performed as previously reported [39]. Quantitative real-time PCRs (qRT-PCRs) were performed in triplicate using the ChamQ supermix (Vazyme, Clinisciences, Roma, Italy) and carried out in the MiniOpticon PCR detection system (Bio-Rad, Milano, Italy). Data were normalized to those for *CDC28* and *CDC34* reference genes and organized with CFX Manager software (Bio-Rad, Milano, Italy); primers are available upon request.

### 2.16. FM4-64 Internalization

*wt[α-syn]* cells were grown overnight to the exponential phase in minimal medium in the absence or presence of 0.5% fungal extracts. Cells were pelleted, resuspended in 100 μL of fresh medium containing 40 μM FM4-64 (Molecular Probes, Eugen, OR, USA) from a stock solution of 4 mM in dimethylsulfoxide, and incubated at 30 °C for 15 min with shaking. Then cells were pelleted, resuspended in fresh medium, and immediately counted at the fluorescent microscope for the presence of the dye at the vacuolar membrane.

### 2.17. In Vitro α-syn Fibrillation

In accordance with previously reported protocols [40], recombinant α-syn (kind gift from Prof. P. Polverino de Laureto) [41] samples (250 μM), filtered with a 0.22 μm pore-size filter (Millipore, Bedford, MA, USA), were incubated at 37 °C in 20 mM sodium phosphate buffer, pH 7.4, up to 7 days under shaking at 900 rpm with a thermo-mixer in the absence (control) or in the presence of *G. frondosa* or *H. erinaceus* by using the mass ratio protein/substance of 1:1 (1x).

### 2.18. Morphological Characterization by Electron Microscopy Imaging

The morphology of α-syn aggregates was investigated by transmission electron microscopy (TEM). A total of 5 μL aliquots of α-syn incubated in the presence or in the absence of *G. frondosa* or *H. erinaceus* extracts were withdrawn at different aggregation times, loaded onto a formvar/carbon-coated 400 mesh nickel grids (Agar Scientific, Stansted, UK) and negatively stained with 2.0% (*w/v*) Tungstic acid (Sigma-Aldrich, St. Louis, MO, USA). The grid was air-dried and examined using a JEM 1010 transmission electron microscope at 80 kV excitation voltage.

### 2.19. Thioflavin T (ThT) Assay

The ThT binding assay was performed according to LeVine [42], using a 25 µM ThT solution in 20 mM sodium phosphate buffer, pH 7.0. Each sample, diluted at a final concentration of 6.25 µM, was transferred into a 96-well half-area, low-binding, clear bottom (200 μL/well); and ThT fluorescence was read at the maximum intensity of fluorescence of 485 nm using a Biotek Synergy 1H plate reader; buffer fluorescence was subtracted from the fluorescence values of all samples. In the control experiments, a significant interference of the highest concentration of *G. frondosa* or *H. erinaceus* on ThT fluorescence was observed, so the mass ratio protein: extract with lowest fluorescence interference was selected (Supplementary Figure S2). We performed two different controls to evaluate the effects of: (i) extracts on ThT fluorescence (Supplementary Figure S2A); (ii) extracts on α-syn-ThT complex, adding *G. frondosa* or *H. erinaceus* at two mass ratios protein: extract (1× and 2×) in order to assess the possible quenching effects (Supplementary Figure S2B). The highest dose interferes with the experimental procedure increasing the fluorescence of the ThT probe and interfering with the α-syn-ThT complex fluorescence, so we used the lowest mass ratio 1x to better discriminate the extracts effects on the α-syn amyloid aggregation process.

### 2.20. Fly Stocks and Husbandry

*Drosophila melanogaster* stocks, obtained from Bloomington Drosophila Stock Centre (Indiana University, Bloomington, IN, USA), were allowed to develop under a 12:12 h light:dark cycle at 25 °C and 60% (±5%) relative humidity. The flies were kept in plastic vials containing standard *Drosophila* food (Formula 4–24^®^ media, Carolina Biological, Burlington, NC, USA) and yeast. Crosses were set up using virgin females of UAS-αSyn line (#8146) that were mated to males of the nsyb-GAL4 driver line (#39171). The progeny expressed human α-synuclein in the neurons and the flies were referred to as PD flies. PD flies were kept at 27 °C to enhance GAL4-driven expression of the UAS-αSyn constructs.

### 2.21. Drosophila melanogaster Longevity Assay

After eclosion, male and female PD flies were separated according to the sex every 24 h [43]. A total of nearly 1000 male and 1000 female PD flies were randomly divided into 4 groups: Control (CNT) 0.2%, *G. frondosa* extract (G.f.) 0.2%, CNT 0.05%, and G.f. 0.05%. The extract of *G. frondosa* was diluted in the water that served to soak the culture medium and flies were supplemented lifelong. Every 3-4 days, the flies were transferred into vials containing fresh food and the number of dead flies was counted. This was repeated until all flies died. Kaplan–Meier survival curves were generated for lifespan assessment through OASIS2 software [44].

### 2.22. Western Blot Analysis of D. melanogaster Heads

Approximately 20 heads of PD flies supplemented or not with G.f. 0.05% for 25 days were homogenized in 7.0 M urea, 2 M thiourea, 4% CHAPS, 60 mM dithiothreitol. Samples were diluted in Laemmli buffer containing 5% β-mercaptoethanol. 15 µg of protein extract was loaded per lane and run in 13% polyacrylamide gels using a mini-Protean Tetracell (Biorad, Hercules, CA, USA) and transferred onto nitrocellulose membranes (0.2 μm) using a Trans-Blot Turbo transfer system (Biorad, Hercules, CA, USA) as previously described [45]. Anti-α-Synuclein XP^®^ Rabbit (#51510 Cell Signaling Technology, Beverly, MA, USA) antibody was used at 1:1,000 dilution. Moreover, an anti-β-actin (1:5000, #MA1-744, Thermo Fisher Scientific, Waltham, MA, USA) was used for internal normalization. HRP-goat anti-rabbit secondary antibody was used at 1:10,000 dilution. Immunoblots were developed using the enhanced chemiluminescence detection system (ECL). The chemiluminescent images were acquired using LAS4010 (GE Health Care Europe, Upsala, Sweden). The immunoreactive specific bands were quantified using Image Quant-L software.

## 3. Results

### 3.1. Analysis of Fungal Extract Composition

**Figure 1:**
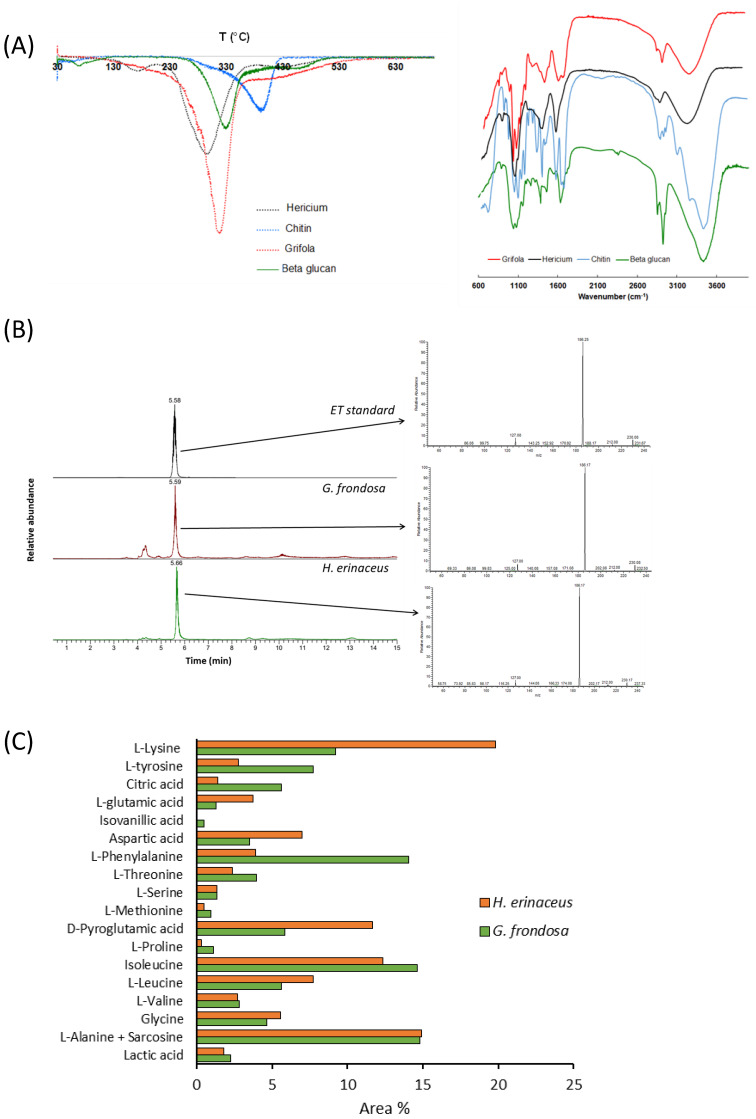
Chemical analysis of fungal extracts (**A**) DTGA curves (left) and FT-IR spectra (right) of the lyophilized aqueous extracts of *G. frondosa* and *H. erinaceus* fruiting bodies, and of β-glucan and chitin standards. (**B**) LC/MS (left) and MS/MS spectra of ergothioneine (ET) obtained by fragmentation of the ion at *m*/*z* 230 for the aqueous extracts of *G. frondosa* and *H. erinaceus* fruiting bodies and an ET standard. (**C**) Relative percentage of metabolites in both *G. frondosa* and *H. erinaceus*.

The aqueous extracts of *G. frondosa* and *H. erinaceus* were first lyophilized and then analyzed with different methods to evaluate their composition. The results obtained by TGA first derivatives (DTGA, on the left) and FT-IR (on the right) of the two fungal extracts, and of the β-glucan and chitin standards, are reported in Figure 1A.

According to the literature [46], three main stages of thermal degradation could be identified for fungal extracts. The first one in the range 120–250 °C, more evident for *H. erinaceus* than for *G. frondosa*, is associated with the degradation of side chain polysaccharides, such as α-glucans or glycoproteins [47,48], that occurs at a lower temperature in comparison to polysaccharide backbone degradation [49,50]. In the second stage (250–350 °C) the major weight loss occurs and is related to β-glucan thermal degradation, as confirmed by the good overlapping with the standard signal and in accordance with the literature [46,47]. As expected, β-glucans content was 66.7% and 61.7 % for *G. frondosa* and *H. erinaceus,* respectively. The weight loss observed in the third stage (350–500 °C) could be reasonably attributed to chitin-glucans and pure chitin thermal degradation (Figure 1A, left panel), as also previously reported [51].

As regards FT-IR characterization, both samples showed bands in the regions characteristic for chitin and β-glucan [25,26,52,53,54,55], confirming the results obtained by DTGA analysis (Figure 1A, right panel).

Ergothioneine was identified through mass spectrometry, by fragmentation of the ion at *m*/*z* 230, which typically shows fragment ions at *m*/*z* 186 and *m*/*z* 127 (Figure 1B). Both samples contained ergothioneine that was quantified by LC/MS analysis in SIM mode and was 1.31 mg/g and 0.99 mg/g in the extracts of *H. erinaceus* and *G. frondosa*, respectively (Figure 1C, Supplementary Figure S1).

Free amino acids, as well as unsaturated fatty acids, ergosterol, and other compounds were investigated by GC/MS analyses (Supplementary Table S1). Both the samples were good sources of amino acids (including essential ones), as well as of unsaturated fatty acids (Table 1), in agreement with previously reported data (see references in Table 1).

A strict comparison between the samples revealed that *G. frondosa* extract contained some molecules absent in *H. erinaceus* extract, such as adipic acid, isovanillic acid, azelaic acid, and stearic acid. The results reported in Figure 1C, expressed as the relative percentage of each amino acid with respect to the total, allow us to highlight some differences between the two samples: L-glutamic acid, L-lysine, aspartic acid, and D-pyroglutamic acid were more abundant in *H. erinaceus* than in *G. frondosa* extract, whereas this latter was richer in L-tyrosine, citric acid, and L-phenylalanine.

Differences were also found in the monosaccharide composition of the two samples. The main monosaccharide composition of the *G. frondosa* extract consisted of 40% mannose (identified as 1,2,3,4-tetramethylmannose) and 60% galactopyranose (identified as 2,3,6,-tri-O-methyl-d-galactopyranose) with traces of trehalose (recognized as D-(+) trehalose, pentamethyl), dulcitol (recognized as dulcitol, hexamethyl ether) and free glucose (identified as D-glucose,2,3,4,5-tetra-O-methyl). Unlikely, the more abundant monosaccharides of the *H. erinaceus* extract were arabitol (80%, identified as L-(-)-arabitol, pentamethyl ether) and glucose (20%, identified as D-glucose, 2,3,4,6-tetra-O-methyl) with traces of dulcitol (identified as dulcitol, hexamethyl ether).

Therefore, β-glucans represent about two thirds of the total aqueous extracts, while the remaining components are chitin, amino acids, unsaturated fatty acids, and monosaccharides.

### 3.2. Fungal Extracts Extend Chronological Lifespan of Yeast Cells

**Figure 2:**
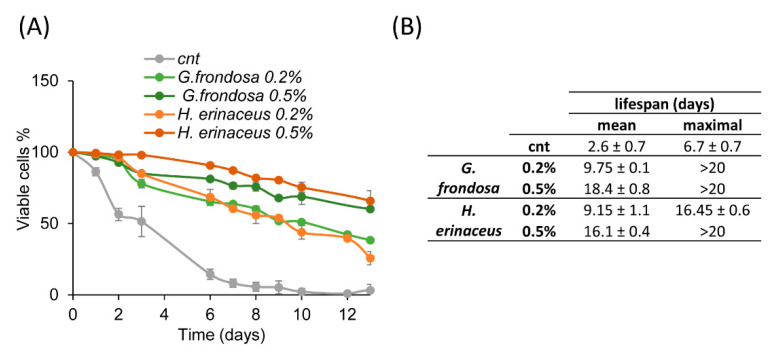
Fungal extracts extend yeast lifespan. (**A**) CLS of wt cells grown in minimal medium containing 2% glucose in the absence (cnt) or presence of 0.2% or 0.5% extract of *G. frondosa* or *H. erinaceus*. (**B**) Mean and maximal lifespan of wt cells grown in the presence of the indicated fungal extracts.

In order to evaluate whether *G. frondosa* and *H. erinaceus* extracts could exert anti-aging effects, they were added to exponentially growing wild type yeast cells. Fungal extracts were tested at two different concentrations (0.2% or 0.5%) in a synthetic minimal medium. Results show that both extracts prolonged the chronological lifespan (CLS) of yeast cells in a similar way, with a dose-dependent response (Figure 2A,B). In fact, the mean lifespan resulted three times longer in cells treated with 0.2% extracts and increased up to 5 times with 0.5% extracts (Figure 2B).

### 3.3. The Fungal Extracts Inhibit the Ras/PKA Pathway

**Figure 3:**
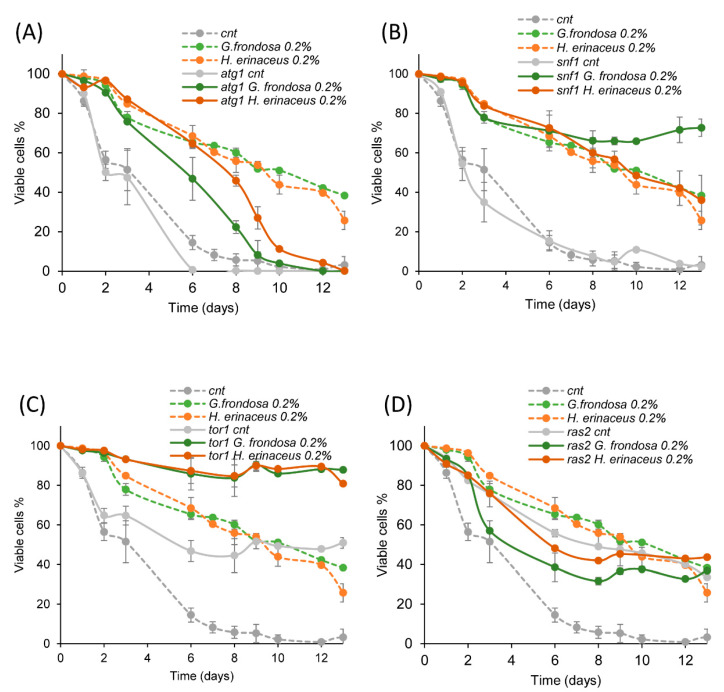
Ras pathway is required to extend yeast lifespan upon fungal treatment. (**A**–**D**) CLS of wt (cnt) and (**A**) *atg1*Δ, (**B**) *snf1*Δ, (**C**) *tor1*Δ, (**D**) *ras2*Δ cells, grown in minimal medium containing 2% glucose in the absence or presence of 0.2% *G. frondosa* or *H. erinaceus* extracts. Curves of wt untreated cells (cnt) and treated with the extracts were repeated in (**A**–**D**) (dashed lines).

In *S. cerevisiae,* different signaling pathways are able to modulate the yeast lifespan. In particular, the Snf1/AMPK and the autophagy pathways have anti-aging effects, while the TORC1 and the Ras/PKA pathways exert pro-aging effects [2]. To identify pathways involved in the extension of CLS induced by fungal extracts, we tested their effects on yeast strains bearing a deletion inactivating one of the aforementioned pathways (*snf1Δ*, *atg1Δ*, *tor1Δ*, *ras2Δ*). The lifespan extension was still clearly visible in *snf1Δ* (Figure 3A), *atg1Δ* (Figure 3B) and *tor1Δ* (Figure 3C) strains treated with 0.2% *G. frondosa* or *H. erinaceus* extracts. On the contrary, the lifespan of cells lacking *RAS2* was not affected by either fungal extracts (Figure 3D), indicating that the Ras/PKA pathway was required to mediate the anti-aging effect of these extracts.

**Figure 4:**
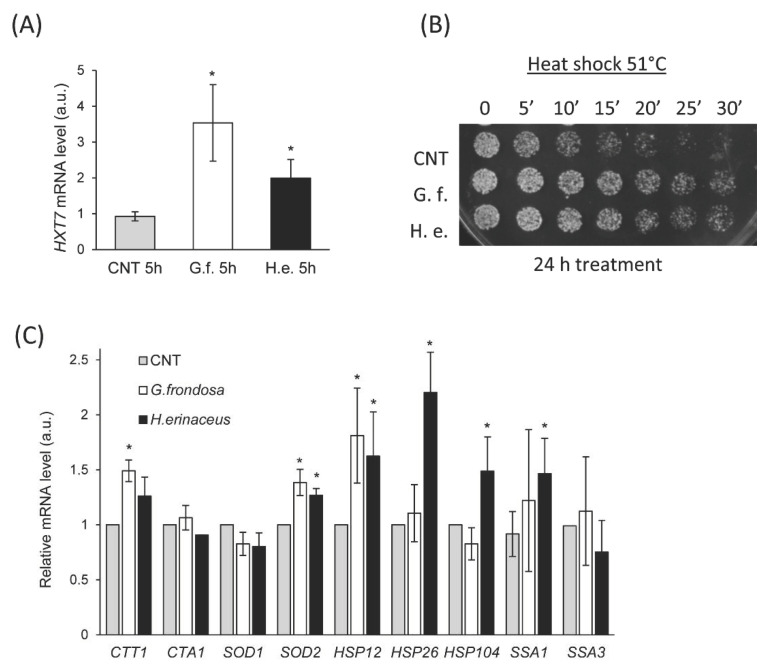
Fungal extracts inhibit the Ras/PKA pathway. (**A**) Real time PCR quantification of *HXT7* mRNA in cells treated or not with 0.5% extract of *G. frondosa* or *H. erinaceus* for 5 h. (**B**) Drop test on YPD plates of wt cells (CNT) treated or not with 0.5% extract of *G. frondosa* or *H. erinaceus* for 24 h, after heat shock at 51 °C for the indicated time. (**C**) Real time PCR quantification of mRNAs of the indicated genes encoding for heat shock proteins in cells treated or not with 0.5% extract of *G. frondosa* or *H. erinaceus* for 24 h. * *p* < 0.05.

The Ras/PKA pathway regulates several aspects of cellular physiology, such as growth, response to glucose and stress conditions [67]. This is achieved through phosphorylation of different transcription factors, often co-regulated by other signaling pathways. Recently, we showed that the expression level of the *HXT7* gene can be used as a reporter of Ras/PKA activity [68], being repressed when the pathway is active (*i.e.,* in high glucose conditions in the exponential phase of growth). Thus, to evaluate the effect of fungal extracts of the Ras/PKA pathway, we measured the expression level of *HXT7* in cells treated with 0.5% fungal extracts for 5 h (Figure 4A). In agreement with CLS results showing that *RAS2* is essential to promote a lifespan extension (Figure 3D), we found that *HXT7* was de-repressed upon treatment with *G. frondosa* or *H. erinaceus* extracts (Figure 4A), supporting the hypothesis that fungal extracts inhibit the Ras/PKA pathway. Another typical hallmark of PKA activity impairment is a higher resistance to heat shock [69], thus we tested the ability of cells treated with fungal extracts to survive upon heat shock at 51 °C (Figure 4B). Although 5 h treatment did not alter heat resistance (data not shown), 24 h treatment with 0.5% fungal extracts strongly increased survival upon heat shock (Figure 4B).

This is consistent with an increased expression of genes encoding for the heat shock proteins Hsp12, Hsp26, and Ssa1, whose expression is regulated by the transcription factor Hsf1, controlled by the Ras/PKA pathway [67]. On the contrary, no differences were observed in the expression of *HSP104* and of *SSA3* (encoding for an isoform of Hsp70), (Figure 4C), suggesting that there is no general induction of chaperones.

Moreover, the Ras/PKA pathway regulates the redox response. The treatment with both mushroom extracts induced a significant upregulation on *CTT1* and *SOD2* expression, encoding for cytoplasmic catalase T and mitochondrial superoxide dismutase 2, respectively (Figure 4C), in line with the inhibitory effect of the extracts on this pathway.

Altogether, these data indicate that fungal extracts are able to repress the Ras/PKA pathway, thus resulting in both increased lifespan and response to stress.

### 3.4. Fungal Extracts Reduce α-Synuclein Toxicity in Yeast

**Figure 5:**
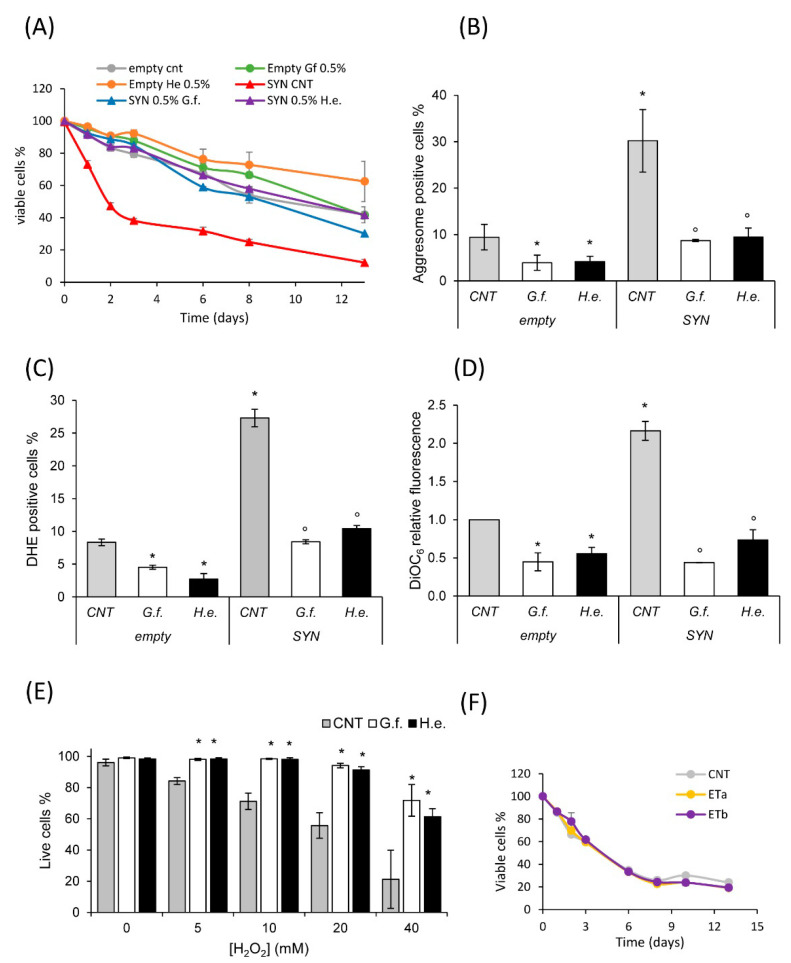
Fungal extracts reduce α-synuclein toxicity. (**A**) CLS of *wt[empty]* and *wt[αSyn]* cells grown in minimal medium containing 2% glucose in the absence or presence of 0.5% extract of *G. frondosa* or *H. erinaceus*. (**B**) Aggresome content (assayed by the PROTEOSTAT Aggresome kit) of *wt[empty] and wt[αSyn]* cells grown for 24 h in the absence or presence of 0.5% extract of *G. frondosa* or *H. erinaceus*. (**C**) ROS content (assayed by DHE staining) of *wt[empty]* and *wt[αSyn]* cells grown for 24 h in the absence or presence of 0.5% extract of *G. frondosa* or *H. erinaceus*. (**D**) Mitochondrial membrane potential (assayed by DiOC_6_ staining) of *wt[empty] and wt[αSyn]* cells grown for 24 h in the absence or presence of 0.5% extract of *G. frondosa* or *H. erinaceus*. (**B**–**D**) Mean ± standard deviations are shown. * *p* < 0.05 relative to control *wt[empty]* cells***,*** ° *p* < 0.05 relative to control *wt[αSyn]* cells. (**E**) *wt[αSyn]* cells were grown in minimal medium containing 2% glucose in the absence or presence of 0.5% extract of *G. frondosa* or *H. erinaceus* for 24 h. Cells in exponential phase of growth were treated with the indicated concentrations of H_2_O_2_ for 4 h and cell viability was assayed with propidium iodide staining by cytofluorimetric analysis. Mean ± standard deviations are shown. * *p* < 0.05 relative to the same concentration of H_2_O_2_ in cells. (**F**) (A) CLS of *wt[αSyn]* cells grown in minimal medium containing 2% glucose in the absence or presence of ergothioneine (ET) at the following concentrations: a = 0.0005%, b = 0.00075%.

Synucleinopathies, like Parkinson’s Disease (PD), are associated with α-synuclein (α-syn) misfolding [7,8,9] and heterologous expression of α-syn in budding yeast has been extensively used as a model of these pathologies [70]. Yeast cells overexpressing human α-syn present a significant shortening of the lifespan compared to control cells bearing the empty plasmid ([5], Figure 5A). Importantly, treatment with 0.5% extract of both fungi increased the lifespan of α-syn-expressing cells (Figure 5A).

α-syn toxicity is associated with its misfolding and aggregation, both in human cells and in yeast [71]. Then, the level of aggresomes was measured in control cells and in cells overexpressing α-syn, to evaluate whether *G. frondosa* and *H. erinaceus* extracts could affect protein aggregation. Strikingly, the fluorescence of an aggresome dye, proportional to the level of protein aggregation in cells, was strongly reduced after 24 h treatment with fungal extracts in control cells (empty vector, Figure 5B). This effect was particularly evident in cells expressing α-syn, whose level of aggresomes was three-fold higher than that observed in control cells, but reached the level of control cells after 24 h treatment with the mushroom extracts (Figure 5B). Other features of α-syn toxicity are high levels of reactive oxygen species (ROS) and mitochondrial dysfunction, which contribute to reduce the cellular lifespan. Remarkably, treatment with fungal extracts significantly reduced the ROS level (Figure 4C), and mitochondrial membrane potential (Figure 5D), especially in cells overexpressing α-syn. In line with these data, and with the observed induction of genes involved in redox response (Figure 4C), an increased dose-dependent resistance to H_2_O_2_ treatment was displayed by cells overexpressing α-syn treated with fungal extracts (Figure 5E).

Considering that ET is a potent antioxidant agent, we tested whether the observed effect could be due to the presence of this compound in both extracts. Pure ET was added to α-syn expressing cells at two different doses (0.0005% and 0.00075%), mimicking the abundance of this compound in the two mushroom extracts (about 1 and 1.5 mg/g of ET in 0.5% treatments). Surprisingly, ET had no effect on the lifespan of cells at the concentrations tested (Figure 5F), suggesting that the anti-aging effect of mushroom extracts is either mediated by other components and/or by a synergistic interaction between the bioactive molecules of the extracts.

**Figure 6:**
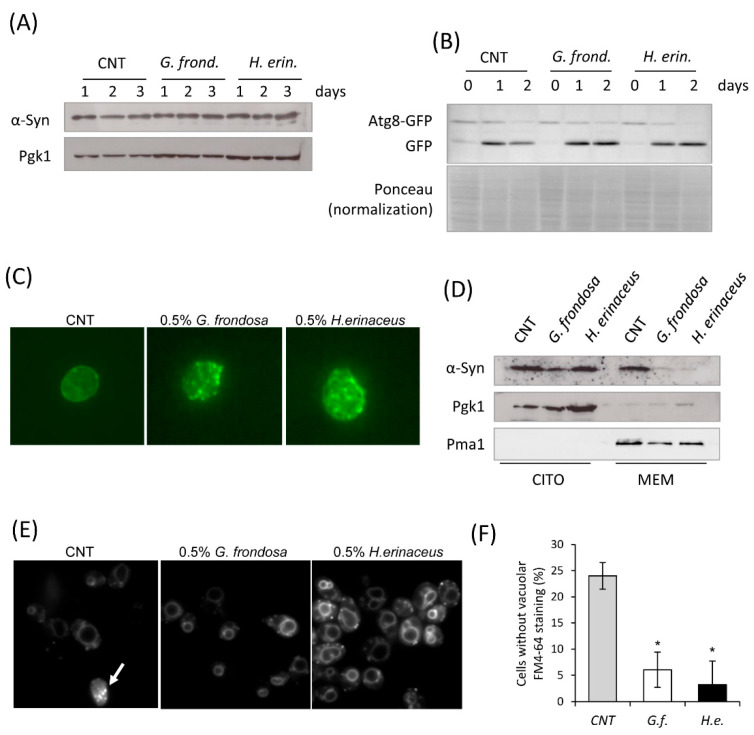
Fungal extracts induce α-synuclein delocalization. (**A**) Western analysis using anti-α-synuclein antibody on total extracts from *wt[αSyn]* cells after 24 h treatment with 0.5% fungal extract. Pgk1 was used as loading control. (**B**) Western analysis using anti-GFP antibody on total extracts from *wt[αSyn][Atg8-GFP]* cells treated with 0.5% fungal extract. PonceauS staining was used as loading control. (**C**) Immunofluorescence showing localization of α-synuclein in cells untreated or treated for 1 day with 0.5% fungal extracts. (**D**) Western analysis using anti-α-synuclein antibody on cytoplasmic and membrane fractions isolated from *wt[αSyn]* cells after 24 h treatment with 0.5% fungi extract. Pgk1 was used as cytoplasmic marker, Pma1 as membrane marker. (**E**,**F**) *wt[αSyn]* cells were grown in exponential phase in the presence or absence of 0.5% extract of *G. frondosa* or *H. erinaceus* and stained for 15 min with FM4-64, as reported in materials and methods. Cells with or without FM4-64 staining at the vacuolar membrane were counted at the microscope, mean ± standard deviations are shown. * *p* < 0.05 relative to control condition (no extract).

The ubiquitin-proteasome system and autophagy are deeply involved in the clearance of α-syn [3]. However, treatment with fungal extracts did not reduce total α-syn levels, given that α-syn was still present in the cells three days after the exponential phase (Figure 6A). Consistently, the induction of autophagy, clearly evident in the stationary phase by the accumulation of free GFP in cells expressing Atg8-GPF fusion protein, was comparable between untreated and treated cells with 0.5% mushroom extracts (Figure 6B). However, subcellular localization of α-syn was strongly affected by fungal extracts after 24 h treatment, since the level of α-syn localized at the plasma membrane, detected by immunofluorescence (Figure 6C) and found in the membrane fraction by immunoblot, was significantly reduced (Figure 6D). Cellular localization of α-syn is known to be associated with its toxicity [72], therefore the decreased α-syn toxicity in the presence of mushroom extracts could be linked to its reduced localization in the plasma membrane, in agreement with the reduced toxicity of the α-syn A30P mutation in yeast cells, which impairs the binding of α-syn to membranes [5].

To support this hypothesis, we tested the toxicity of α-syn on membrane activities by monitoring the internalization of the fluorescent vital dye FM4-64, which can be used to study membrane internalization and transport to the vacuole [73]. In cells expressing α-syn, FM4-64 localization at the vacuolar membrane was defective, as previously reported [72], since 25% of cells did not exhibit any vacuolar staining. On the contrary, in cells previously treated with both fungal extracts, FM4-64 was rapidly internalized and accumulated at the vacuolar membrane in almost all cells, indicating that membrane internalization and transport to the vacuole was fully restored in α-syn expressing cells (Figure 6E,F).

Altogether these data indicate that fungal extracts reduce α-syn toxicity through different processes, such as the decrease of protein aggregation, reduction of the ROS level, and α-syn membrane delocalization.

### 3.5. Fungal Extracts Inhibit α-Synuclein Aggregation In Vitro

**Figure 7:**
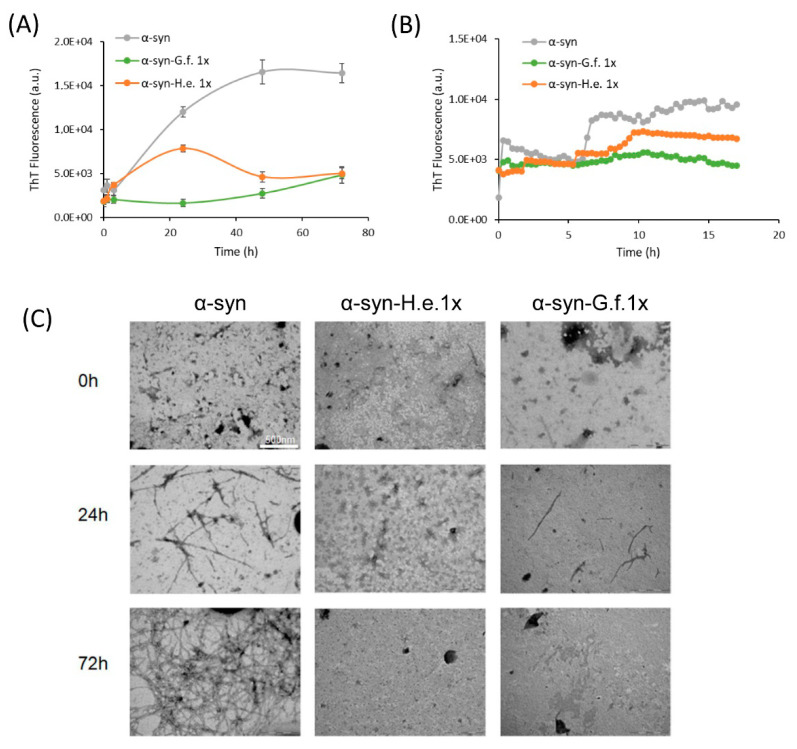
Fungal extract effects on α-syn aggregation process. (**A**) ThT fluorescence intensity of α-syn samples aggregated in the absence (α-syn) or in the presence of *G. frondosa* (*a*-syn-G.f.) or *H. erinaceus* (a-syn-H.e.) extract at α-syn:extract mass ratio 1:1 (1x) recorded at different times of aggregation (0 h, 24 h, 48 h and 72 h) or (**B**) in continuous for the first 15 h. (**C**) TEM images of α-syn aggregated for 0 h, 24 h or 72 h in the presence or in the absence of *G. frondosa* or *H. erinaceus* 1×.

The effect of mushroom extracts on α-syn aggregation was also investigated in vitro. We first examined the inhibitory potential of the two extracts on the amyloid aggregation kinetic of α-syn. For this purpose, we used thioflavin assay, ThT, a common analytical fluorescent method, widely used for determining the kinetic of the amyloid self-assembly process, showing a strong increase in ThT fluorescent intensity upon binding to β-sheet-rich supramolecular structures. We monitored the aggregation of monomeric α-syn in the absence and presence of *G. frondosa* or *H. erinaceus* extracts at the α-syn:extract mass ratio of 1:1 (Figure 7A,B).

The results revealed that both extracts successfully and significantly inhibited the self-assembly of α-syn in ThT positive aggregates. To discard a putative interaction between the extract compounds and the ThT dye, which could modulate the fluorescence yield of ThT rather than the inhibition of amyloid formation, both extracts at two different concentrations were added to a ThT solution or to α-syn/ThT solution (Supplementary Figure S2). No significant reduction of ThT fluorescence was observed at the lowest mass ratio used indicating that both *G. frondosa* and *H. erinaceus* extracts specifically hindered the formation of α-syn amyloid structures (Supplementary Figure S2). To more directly monitor the action of *G. frondosa* or *H. erinaceus* extracts, we performed TEM analysis (Figure 7C). Both extracts resulted in a strong reduction of aggregation compared to α-syn alone, in line with the results of the ThT assay.

These data suggest that mushroom extracts can directly inhibit α-syn aggregation also in vitro, further supporting their anti-aggregation properties.

### 3.6. G. frondosa Extract Extends Lifespan in a Drosophila melanogaster Model of Parkinson’s Disease

**Figure 8:**
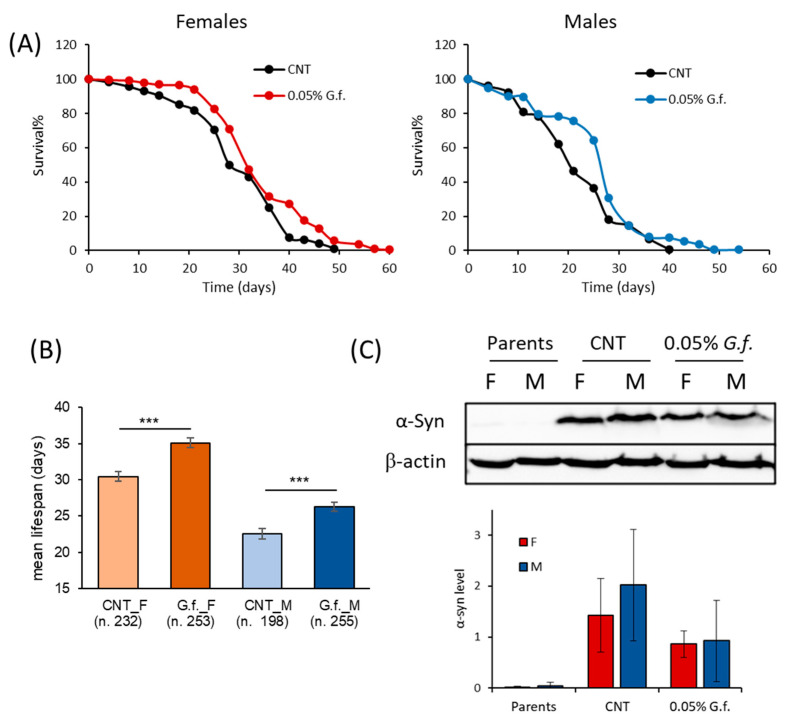
Fungal extract extends the lifespan of adult female and male flies. (**A**) Flies were supplemented with 0.05% *G. frondosa* extract lifelong. Data are presented as a percentage of survival of flies as a function of time (in days), evaluated in ten independent measurements for each group of flies by the Kaplan–Meier estimator (OASIS2 software). (**B**) The Log-rank test (OASIS2 software) was used to compare the mean lifespan of both male and female flies. *** *p* < 0.001 *vs* the respective CNT. (**C**) Western analysis of α-syn expression in fly brains supplemented or not with 0.05% *G. frondosa* extract. Representative immunoblot images and histograms of band-normalized OD are shown. Data are presented as mean ± SEM of three independent experiments.

To investigate the effect of the mushroom extracts in a multicellular organism, a *D. melanogaster* model of PD was used. Considering that the two extracts showed similar effects on the yeast model, we decided to test only *G. frondosa* extract in *D. melanogaster*. To test the possible pro-longevity effect of *G. frondosa* extract, male and female PD flies were maintained on a standard diet supplemented with 0.2% *G. frondosa* lifelong (Supplementary Figure S3). Surprisingly, the mean lifespan of female flies supplemented with 0.2% *G. frondosa* extract was significantly lower in respect to control flies (4% decrease, *p* < 0.0001), while the mean lifespan of male flies was comparable to that of control flies (Supplementary Figure S3). On these bases, we decided to investigate the effect of a lower *G. frondosa* extract concentration (0.05%) on lifespan extension of *D. melanogaster* (Figure 8A). Interestingly, this concentration did not show any toxic effect and significantly increased the mean lifespan of both male and female flies (Figure 8B). In particular, 0.05% *G. frondosa* extract increased the mean lifespan by around 15% (*p* < 0.0001) and 17% (*p* < 0.0001), in females and males, respectively. In addition, maximal longevity increased by 9 (+22.5%) and 14 (+35%) days for females and males, respectively.

To assess the effect of *G. frondosa* extract on α-syn expression, we evaluated the α-syn level in the brain of flies supplemented or not with 0.05% *G. frondosa* extract for 25 days by immunoblot analysis (Figure 8C). As expected, female and male parents before crossing did not express α-syn, while PD flies strongly expressed human α-syn. *G. frondosa* extract induced a reduction, although not statistically significant, of the α-syn level in both male and female flies.

In conclusion, the neuroprotective properties of *G. frondosa* extract were confirmed also using a multicellular model of PD.

## 4. Discussion

It is well known that some medicinal mushrooms improve healthy aging in animal models [20,32], exerting a number of health benefits that may improve human well-being [74]. Here, we prepared aqueous extracts from *G. frondosa* and *H. erinaceus* to evaluate their potential anti-aging and neuroprotective properties.

We showed that in yeast cells, these extracts increased lifespan in a Ras/PKA dependent manner and reduced ROS levels, confirming and extending what was previously reported in other model organisms for *G. frondosa* extracts [24,25]. Furthermore, these extracts reduced α-syn-induced premature aging through multiple mechanisms, i.e., reduction of ROS and the restoration of membrane functionalities together with the reduction of protein aggregation. Strikingly, this effect was also demonstrated in the invertebrate model of PD in *D. melanogaster*, where 0.05% *G. frondosa* extract led to a significant mean lifespan extension, both in male and in female flies. These data corroborate the results obtained in yeast and could be due to the, herein shown, capability of the extract to decrease α-syn aggregation. Interestingly, both mushroom extracts consistently reduced the progression of α-syn fibrillation process in vitro. This can be correlated with the binding of some component of the extracts to a monomer and/or intermediates states of α-syn. The monomer and/or sub- and near-critical oligomers depletion would subsequently lead to the blockage of the aggregation pathway. Nevertheless, we cannot exclude the contribution of other more indirect mechanisms in the reduction of α-syn toxicity in the in vivo models.

Although we did not identify the specific components responsible for the observed effect ―which are likely the result of the synergy among different molecules―we characterized both extracts by chemical analytical techniques. They contained an array of different molecules and metabolites, such as β-glucans, amino acids, fatty acids, ergosterol, ergothioneine, and chitin. On the basis of TGA results (Figure 1A left) β-glucans were present in the two samples *G. frondosa* and *H. erinaceus* in the percentage of 66.7% and 61.7%, respectively.

One of the components of the mushroom extracts is ergothioneine (ET), which is known for its effect on lifespan extension [75,76]. It should be noted that, in our extracts, ET concentration was about 0.1% (1.31 mg/g and 0.99 mg/g in the extracts of *H. erinaceus* and *G. frondosa*, respectively). However, unexpectedly, the lifespan extension induced by mushroom extracts on yeast cells was not mimicked by pure ET (administered at the same concentration present in the extracts), suggesting that in yeast cells ET alone is not responsible for this effect. *H. erinaceus* also contains diterpenoids with interesting neuroprotective effects [18]. However, considering their low water solubility (they are usually extracted with ethanol/methanol procedures) they are unlikely to be responsible for the observed activities exerted by our aqueous extracts.

Some differences exist between the two extracts in the relative proportion of the amino acid composition. The amount of L-glutamic acid, aspartic acid, and D-pyroglutamic acid is higher in *H. erinaceus* than in *G. frondosa*, whereas the latter is richer in L-tyrosine, citric acid, and L-phenylalanine. However, large variations were described in the amount of each amino acid in *G. frondosa* [15,61], reflecting different extraction procedures and the cultivation substrate, which could affect the variety and the amount of amino acids [15]. In addition, lactic acid, sarcosine, D-pyroglutamic acid, adipic acid, crotonic acid, glyceric acid, and azelaic acid have also been detected both in *G. frondosa* and *H. erinaceus* extracts, whereas L-valine, L-proline, citric acid, and L-tyrosine have been detected only in *G. frondosa* extract and isovanillic acid, palmitic acid, and linoelaidic acid only in *H. erinaceus* extract. It is however difficult to draw conclusions on the amino acids profile of the extracts, given that the role of amino acids on longevity is still controversial and puzzling. For example, lowering the intake of specific amino acids such as serine, threonine, and valine can be beneficial and extend the lifespan [77,78], while an increase in glutamate seems to exert an anti-aging effect [79].

In conclusion, the data presented here may have a great deal of implications and form the basis of future studies to elucidate the effects of mushroom-mediated inhibition of the α-syn aggregation process at the molecular level. Moreover, this study opens interesting avenues to set up further strategies and to have better insight regarding mechanisms involved in deciphering the role of mushroom extracts in the inhibition of amyloid formation and in the prevention/alleviation of synucleinopathies, as well as potentially in other protein misfolding-related diseases.

## Figures and Tables

**Table 1:** Analytes identified by GC/MS and comparison with bibliographic references.

Analyte	*H. erinaceus*	Reference (mg/g) Fruiting Body	*G. frondosa*	Reference (mg/g) Fruiting Body
Lactic acid	D	-	D	-
L-Alanine	D	2.43 [56] 0.11 [57] D [58] D [59]	D	2.15 [60] 3.13 [60] 5.22 [61] 2.77 [56]
Sarcosine	D	-	D	-
Glycine	D	1.03 [56] 0.11 [57] D [58]	D	1.53 [60] 1.53 [60] 2.46 [61] 0.57 [56]
L-Valine	D	0.30 [56] 0.10 [57] D [58]	D	-
L-Leucine	D	2.38 [56] 0.14 [57] D [58]	D	0.05 [60] 0.,09 [60] 0.27 [61] 0.35 [56]
Isoleucine	D	0.07 [57] D [58]	D	0.12 [60] 0.12 [60] 0.56 [61] 0.33 [56]
L-Proline	D	0.10 [57] D [58]	D	-
D-Pyroglutamic acid	D	-	D	-
L-Methionine	D	1.08 [56] 0.03 [57] D [58]	D	4.5 [61] 1.4 [56]
Adipic acid	-	-	D	-
L-Serine	D	0.35 [56] 0.11 [57] D [58]	D	2.91 [60] 2.82 [60] 2.01 [61] 0.97 [56]
L-Lysine	D	0.47 [56] 0.28 [57] D [58] D [59]	D	1.56 [60] 1.28 [60] 5.70 [61] 1.11 [56]
L-Threonine	D	0.78 [56] 0.12 [57] D [58]	D	1.43 [60] 1.44 [60] 4.4 [56]
L-Phenylalanine	D	0.20 [56] 0.05 [57] D [58]	D	0.26 [60] 0.28 [60] 2.71 [61] 0.8 [56]
Aspartic acid	D	0.50 [56] 0.23 [57] D [58]	D	1.61 [60] 1.25 [60] 1.66 [61] 0.64 [56]
Isovanillic acid	-	-	D	1.61 [60] 1.25 [60] 1.88 [61] 0.42 [56]
L-Glutamic acid	D	0.50 [56] 0.42 [57] D [58] D [59]	D	8.01 [60] 9.10 [60] 12.62 [61] 0.67 [56]
Citric acid	D	D [62] D [59]	D	-
L-Tyrosine	D	0.14 [57] D [58] D [62]	D	-
Crotonic acid	D	-	D	-
Glyceric acid	D	-	D	-
Palmitic acid	D	-	D	D [63]
Azelaic acid	-	-	D	-
Linoelaidic acid	D	-	D	D [63]
Oleic acid	D	D [59]	D	D [63]
Stearic acid	-	-	D	-
Ergosterol	D	2.58 [64] 2.72 [64] 2.45 [64] D [65]	D	D [66]

D = Detected.

## Data Availability

The data presented in this study are available on request from the corresponding author.

## Supplementary Materials

The following supporting information can be downloaded at https://www.mdpi.com/article/10.3390/nu14204368/s1. Table S1. Analytes identified by GC/MS on the basis of match with NIST2014 library and the corresponding target ions used to quantify them. Table S2. Yeast strains used in this study. Figure S1 Calibration curve and linear regression curve for ET. Figure S2 Evaluation of interference of fungal extracts on ThT assay. Figure S3. High fungal extract concentrations are toxic for adult flies.

## References

[1] Ruetenik A., Barrientos A. (2018). Exploiting Post-Mitotic Yeast Cultures to Model Neurodegeneration. Front. Mol. Neurosci..

[2] Longo V.D., Shadel G.S., Kaeberlein M., Kennedy B. (2012). Replicative and Chronological Aging in Saccharomyces Cerevisiae. Cell Metab..

[3] Sampaio-Marques B., Pereira H., Santos A.R., Teixeira A., Ludovico P. (2018). Caloric Restriction Rescues Yeast Cells from Alpha-Synuclein Toxicity through Autophagic Control of Proteostasis. Aging.

[4] Sampaio-Marques B., Guedes A., Vasilevskiy I., Gonçalves S., Outeiro T.F., Winderickx J., Burhans W.C., Ludovico P. (2019). α-Synuclein Toxicity in Yeast and Human Cells Is Caused by Cell Cycle Re-Entry and Autophagy Degradation of Ribonucleotide Reductase 1. Aging Cell.

[5] Sampaio-Marques B., Felgueiras C., Silva A., Rodrigues M., Tenreiro S., Franssens V., Reichert A.S., Outeiro T.F., Winderickx J., Ludovico P. (2012). SNCA (α-Synuclein)-Induced Toxicity in Yeast Cells Is Dependent on Sir2-Mediated Mitophagy. Autophagy.

[6] Tenreiro S., Franssens V., Winderickx J., Outeiro T.F. (2017). Yeast Models of Parkinson’s Disease-Associated Molecular Pathologies. Curr. Opin. Genet. Dev..

[7] Dodel R., Csoti I., Ebersbach G., Fuchs G., Hahne M., Kuhn W., Oechsner M., Jost W., Reichmann H., Schulz J.B. (2008). Lewy Body Dementia and Parkinson’s Disease with Dementia. J. Neurol..

[8] Van Den Eeden S.K., Tanner C.M., Bernstein A.L., Fross R.D., Leimpeter A., Bloch D.A., Nelson L.M. (2003). Incidence of Parkinson’s Disease: Variation by Age, Gender, and Race/Ethnicity. Am. J. Epidemiol..

[9] de Rijk M.C., Launer L.J., Berger K., Breteler M.M., Dartigues J.F., Baldereschi M., Fratiglioni L., Lobo A., Martinez-Lage J., Trenkwalder C. (2000). Prevalence of Parkinson’s Disease in Europe: A Collaborative Study of Population-Based Cohorts. Neurologic Diseases in the Elderly Research Group. Neurology.

[10] Reiter L.T., Potocki L., Chien S., Gribskov M., Bier E. (2001). A Systematic Analysis of Human Disease-Associated Gene Sequences in Drosophila Melanogaster. Genome Res..

[11] Feany M.B., Bender W.W. (2000). A Drosophila Model of Parkinson’s Disease. Nature.

[12] Sabaratnam V., Kah-Hui W., Naidu M., David P. (2013). Neuronal Health—Can Culinary and Medicinal Mushrooms Help?. J. Tradit. Complement. Med..

[13] Wasser S.P. (2014). Medicinal Mushroom Science: Current Perspectives, Advances, Evidences, and Challenges. Biomed. J..

[14] Venturella G., Ferraro V., Cirlincione F., Gargano M.L. (2021). Medicinal Mushrooms: Bioactive Compounds, Use, and Clinical Trials. Int. J. Mol. Sci..

[15] Wu J.Y., Siu K.C., Geng P. (2021). Bioactive Ingredients and Medicinal Values of Grifola Frondosa (Maitake). Foods.

[16] Rossi P., Difrancia R., Quagliariello V., Savino E., Tralongo P., Randazzo C.L., Berretta M. (2018). B-Glucans from Grifola Frondosa and Ganoderma Lucidum in Breast Cancer: An Example of Complementary and Integrative Medicine. Oncotarget.

[17] Li I.C., Lee L.Y., Tzeng T.T., Chen W.P., Chen Y.P., Shiao Y.J., Chen C.C. (2018). Neurohealth Properties of Hericium Erinaceus Mycelia Enriched with Erinacines. Behav. Neurol..

[18] Friedman M. (2015). Chemistry, Nutrition, and Health-Promoting Properties of Hericium Erinaceus (Lion’s Mane) Mushroom Fruiting Bodies and Mycelia and Their Bioactive Compounds. J. Agric. Food Chem..

[19] Ratto D., Corana F., Mannucci B., Priori E.C., Cobelli F., Roda E., Ferrari B., Occhinegro A., Di Iorio C., De Luca F. (2019). Hericium Erinaceus Improves Recognition Memory and Induces Hippocampal and Cerebellar Neurogenesis in Frail Mice during Aging. Nutrients.

[20] Vigna L., Morelli F., Agnelli G.M., Napolitano F., Ratto D., Occhinegro A., Di Iorio C., Savino E., Girometta C., Brandalise F. (2019). Hericium Erinaceus Improves Mood and Sleep Disorders in Patients Affected by Overweight or Obesity: Could Circulating Pro-BDNF and BDNF Be Potential Biomarkers?. Evid. Based. Complement. Alternat. Med..

[21] Corana F., Cesaroni V., Mannucci B., Baiguera R.M., Picco A.M., Savino E., Ratto D., Perini C., Kawagishi H., Girometta C.E. (2019). Array of Metabolites in Italian Hericium Erinaceus Mycelium, Primordium, and Sporophore. Molecules.

[22] Trovato Salinaro A., Pennisi M., Di Paola R., Scuto M., Crupi R., Cambria M.T., Ontario M.L., Tomasello M., Uva M., Maiolino L. (2018). Neuroinflammation and Neurohormesis in the Pathogenesis of Alzheimer’s Disease and Alzheimer-Linked Pathologies: Modulation by Nutritional Mushrooms. Immun. Ageing.

[23] Lee L.Y., Chou W., Chen W.P., Wang M.F., Chen Y.J., Chen C.C., Tung K.C. (2021). Erinacine A-Enriched Hericium Erinaceus Mycelium Delays Progression of Age-Related Cognitive Decline in Senescence Accelerated Mouse Prone 8 (SAMP8) Mice. Nutrients.

[24] Song L., Zhou Y., Ni S., Wang X., Yuan J., Zhang Y., Zhang S. (2020). Dietary Intake of β-Glucans Can Prolong Lifespan and Exert an Antioxidant Action on Aged Fish Nothobranchius Guentheri. Rejuvenation Res..

[25] Aranaz P., Peña A., Vettorazzi A., Fabra M.J., Martínez-Abad A., López-Rubio A., Pera J., Parladé J., Castellari M., Milagro F.I. (2021). Grifola Frondosa (Maitake) Extract Reduces Fat Accumulation and Improves Health Span in C. Elegans through the DAF-16/FOXO and SKN-1/NRF2 Signalling Pathways. Nutrients.

[26] Tomas-hernandez S., Blanco J., Garcia-vallvé S., Pujadas G., Ojeda-montes M.J., Gimeno A., Arola L., Minghetti L., Beltrán-debón R., Mulero M. (2021). Anti-Inflammatory and Immunomodulatory Effects of the Grifola Frondosa Natural Compound o-Orsellinaldehyde on LPS-Challenged Murine Primary Glial Cells. Roles of NF-Κβ and MAPK. Pharmaceutics.

[27] Cheng J.H., Tsai C.L., Lien Y.Y., Lee M.S., Sheu S.C. (2016). High Molecular Weight of Polysaccharides from Hericium Erinaceus against Amyloid Beta-Induced Neurotoxicity. BMC Complement. Altern. Med..

[28] Ciecierska A., Drywień M.E., Hamulka J., Sadkowski T. (2019). Nutraceutical Functions of Beta-Glucans in Human Nutrition. Rocz. Panstw. Zakl. Hig..

[29] La Rosa F., Clerici M., Ratto D., Occhinegro A., Licito A., Romeo M., Di Iorio C., Rossi P. (2018). The Gut-Brain Axis in Alzheimer’s Disease and Omega-3. A Critical Overview of Clinical Trials. Nutrients.

[30] Kalaras M.D., Richie J.P., Calcagnotto A., Beelman R.B. (2017). Mushrooms: A Rich Source of the Antioxidants Ergothioneine and Glutathione. Food Chem..

[31] Beelman R.B., Kalaras M.D., Phillips A.T., Richie J.P. (2020). Is Ergothioneine a “longevity Vitamin” Limited in the American Diet?. J. Nutr. Sci..

[32] Roda E., Priori E.C., Ratto D., De Luca F., Di Iorio C., Angelone P., Locatelli C.A., Desiderio A., Goppa L., Savino E. (2021). Neuroprotective Metabolites of Hericium Erinaceus Promote Neuro-Healthy Aging. Int. J. Mol. Sci..

[33] Roda E., Ratto D., De Luca F., Desiderio A., Ramieri M., Goppa L., Savino E., Bottone M.G., Locatelli C.A., Rossi P. (2022). Searching for a Longevity Food, We Bump into Hericium Erinaceus Primordium Rich in Ergothioneine: The “Longevity Vitamin” Improves Locomotor Performances during Aging. Nutrients.

[34] Gründemann D., Harlfinger S., Golz S., Geerts A., Lazar A., Berkels R., Jung N., Rubbert A., Schömig E. (2005). Discovery of the Ergothioneine Transporter. Proc. Natl. Acad. Sci. USA.

[35] Mythri R.B., Harish G., Bharath M.M. (2012). Therapeutic Potential of Natural Products in Parkinson’s Disease. Recent Pat. Endocr. Metab. Immune Drug Discov..

[36] Cheah I.K., Halliwell B. (2021). Ergothioneine, Recent Developments. Redox Biol..

[37] Kondoh H., Teruya T., Kameda M., Yanagida M. (2022). Decline of Ergothioneine in Frailty and Cognition Impairment. FEBS Lett..

[38] Li Q.Z., Wu D., Zhou S., Liu Y.F., Li Z.P., Feng J., Yang Y. (2016). Structure Elucidation of a Bioactive Polysaccharide from Fruiting Bodies of Hericium Erinaceus in Different Maturation Stages. Carbohydr. Polym..

[39] Pessina S., Tsiarentsyeva V., Busnelli S., Vanoni M., Alberghina L., Coccetti P. (2010). Snf1/AMPK Promotes S-Phase Entrance by Controlling CLB5 Transcription in Budding Yeast. Cell Cycle.

[40] Tripodi F., Lombardi L., Guzzetti L., Panzeri D., Milanesi R., Leri M., Bucciantini M., Angeloni C., Beghelli D., Hrelia S. (2020). Protective Effect of Vigna Unguiculata Extract against Aging and Neurodegeneration. Aging.

[41] Palazzi L., Fongaro B., Leri M., Acquasaliente L., Stefani M., Bucciantini M., Polverino de Laureto P. (2021). Structural Features and Toxicity of α-Synuclein Oligomers Grown in the Presence of DOPAC. Int. J. Mol. Sci..

[42] LeVine H. (1999). Quantification of Beta-Sheet Amyloid Fibril Structures with Thioflavin T. Methods Enzymol..

[43] Piegholdt S., Rimbach G., Wagner A.E. (2016). The Phytoestrogen Prunetin Affects Body Composition and Improves Fitness and Lifespan in Male Drosophila Melanogaster. FASEB J..

[44] Yang J.S., Nam H.J., Seo M., Han S.K., Choi Y., Nam H.G., Lee S.J., Kim S. (2011). OASIS: Online Application for the Survival Analysis of Lifespan Assays Performed in Aging Research. PLoS ONE.

[45] Zallocco L., Giusti L., Ronci M., Mussini A., Trerotola M., Mazzoni M.R., Lucacchini A., Sebastiani L. (2021). Salivary Proteome Changes in Response to Acute Psychological Stress Due to an Oral Exam Simulation in University Students: Effect of an Olfactory Stimulus. Int. J. Mol. Sci..

[46] Girometta C., Dondi D., Baiguera R.M., Bracco F., Branciforti D.S., Buratti S., Lazzaroni S.S.E. (2020). Characterization of Mycelia from Wood-Decay Species by TGA and IR Spectroscopy. Cellulose.

[47] Werner K., Pommer L., Broström M. (2014). Thermal Decomposition of Hemicelluloses. J. Anal. Appl. Pyrolysis.

[48] Puanglek S., Kimura S., Enomoto-Rogers Y., Kabe T., Yoshida M., Wada M., Iwata T. (2016). In Vitro Synthesis of Linear α-1,3-Glucan and Chemical Modification to Ester Derivatives Exhibiting Outstanding Thermal Properties. Sci. Reports.

[49] Prime R.B., Bair H.E., Vyazovkin S., Gallagher P.K., Riga A. (2008). Thermogravimetric Analysis (TGA). Therm. Anal. Polym. Fundam. Appl..

[50] Sun B., Hong W., Aziz H., Li Y. (2012). Diketopyrrolopyrrole-Based Semiconducting Polymer Bearing Thermocleavable Side Chains. J. Mater. Chem..

[51] Ospina Álvarez S.P., Ramírez Cadavid D.A., Escobar Sierra D.M., Ossa Orozco C.P., Rojas Vahos D.F., Zapata Ocampo P., Atehortúa L. (2014). Comparison of Extraction Methods of Chitin from Ganoderma Lucidum Mushroom Obtained in Submerged Culture. Biomed Res. Int..

[52] Dahmane E.M., Taourirte M., Eladlani N., Rhazi M. (2014). Extraction and Characterization of Chitin and Chitosan from Parapenaeus Longirostris from Moroccan Local Sources. Int. J. Polym. Anal. Charact..

[53] Gonzaga M.L.C., Menezes T.M.F., De Souza J.R.R., Ricardo N.M.P.S., Soares S.D.A. (2013). Structural Characterization of β Glucans Isolated from Agaricus Blazei Murill Using NMR and FTIR Spectroscopy. Bioact. Carbohydrates Diet. Fibre.

[54] Przekora A., Benko A., Blazewicz M., Ginalska G. (2016). Hybrid Chitosan/β-1,3-Glucan Matrix of Bone Scaffold Enhances Osteoblast Adhesion, Spreading and Proliferation via Promotion of Serum Protein Adsorption. Biomed. Mater..

[55] Shah A., Ahmad M., Ashwar B.A., Gani A., Masoodi F.A., Wani I.A., Wani S.M., Gani A. (2015). Effect of γ-Irradiation on Structure and Nutraceutical Potential of β-d-Glucan from Barley (Hordeum Vulgare). Int. J. Biol. Macromol..

[56] Mau J.L., Lin H.C., Ma J.T., Song S.F. (2001). Non-Volatile Taste Components of Several Speciality Mushrooms. Food Chem..

[57] Sangtitanu T., Sangtanoo P., Srimongkol P., Saisavoey T., Reamtong O., Karnchanatat A. (2020). Peptides Obtained from Edible Mushrooms: Hericium Erinaceus Offers the Ability to Scavenge Free Radicals and Induce Apoptosis in Lung Cancer Cells in Humans. Food Funct..

[58] Aparicio-Razo M., González-Pérez M. (2020). Analysis of Biomolecules of the Fungus Hericium Erinaceus Through the Theory of Electron Transfer of Quantum Chemistry and Its Relationship with the Primary Amino Acids. WORLD J. Pharm. Pharm. Sci..

[59] Kim S.P., Kang M.Y., Kim J.H., Nam S.H., Friedman M. (2011). Composition and Mechanism of Antitumor Effects of Hericium Erinaceus Mushroom Extracts in Tumor-Bearing Mice. J. Agric. Food Chem..

[60] Tabata T., Yamasaki Y., Ogura T. (2004). Comparison of Chemical Compositions of Maitake (Grifola Frondosa (Fr.) S. F. Gray) Cultivated on Logs and Sawdust Substrate. Food Sci. Technol. Res..

[61] Huang S.J., Tsai S.Y., Lin S.Y., Liang C.H., Mau J.L. (2011). Nonvolatile Taste Components of Culinary-Medicinal Maitake Mushroom, Grifola Frondosa (Dicks.:Fr.) S.F. Gray. Int. J. Med. Mushrooms.

[62] Yang F., Wang H., Feng G., Zhang S., Wang J., Cui L. (2021). Rapid Identification of Chemical Constituents in Hericium Erinaceus Based on LC-MS/MS Metabolomics. J. Food Qual..

[63] Zhang Y., Mills G.L., Nair M.G. (2002). Cyclooxygenase Inhibitory and Antioxidant Compounds from the Mycelia of the Edible Mushroom Grifola Frondosa. J. Agric. Food Chem..

[64] Dai X., Zhang J., Han Z., Zhan Y., Wang Y. (2014). Growth Parameters and Ergosterol Content of Mycelia and Fruit Bodies of Ten Hericium Erinaceus Strains Collected from the Wild in Heilongjiang Province,China. Acta Edulis Fungi.

[65] Kim S. (2020). Antioxidant Compounds for the Inhibition of Enzymatic Browning by Polyphenol Oxidases in the Fruiting Body Extract of the Edible Mushroom Hericium Erinaceus. Foods.

[66] Kawai J., Higuchi Y., Hirota M., Hirasawa N., Mori K. (2018). Ergosterol and Its Derivatives from Grifola Frondosa Inhibit Antigen-Induced Degranulation of RBL-2H3 Cells by Suppressing the Aggregation of High Affinity IgE Receptors. Biosci. Biotechnol. Biochem..

[67] Busti S., Coccetti P., Alberghina L., Vanoni M. (2010). Glucose Signaling-Mediated Coordination of Cell Growth and Cell Cycle in Saccharomyces Cerevisiae. Sensors.

[68] Nicastro R., Tripodi F., Gaggini M., Castoldi A., Reghellin V., Nonnis S., Tedeschi G., Coccetti P. (2015). Snf1 Phosphorylates Adenylate Cyclase and Negatively Regulates Protein Kinase A-Dependent Transcription in Saccharomyces Cerevisiae. J. Biol. Chem..

[69] Belotti F., Tisi R., Paiardi C., Groppi S., Martegani E. (2011). PKA-Dependent Regulation of Cdc25 RasGEF Localization in Budding Yeast. FEBS Lett..

[70] Fruhmann G., Seynnaeve D., Zheng J., Ven K., Molenberghs S., Wilms T., Liu B., Winderickx J., Franssens V. (2017). Yeast Buddies Helping to Unravel the Complexity of Neurodegenerative Disorders. Mech. Ageing Dev..

[71] Lázaro D.F., Pavlou M.A.S., Outeiro T.F. (2017). Cellular Models as Tools for the Study of the Role of Alpha-Synuclein in Parkinson’s Disease. Exp. Neurol..

[72] Outeiro T.F., Lindquist S. (2003). Yeast Cells Provide Insight into Alpha-Synuclein Biology and Pathobiology. Science.

[73] Vida T.A., Emr S.D. (1995). A New Vital Stain for Visualizing Vacuolar Membrane Dynamics and Endocytosis in Yeast. J. Cell Biol..

[74] Yadav D., Negi P.S. (2021). Bioactive Components of Mushrooms: Processing Effects and Health Benefits. Food Res. Int..

[75] Pan H.Y., Ye Z.W., Zheng Q.W., Yun F., Tu M.Z., Hong W.G., Chen B.X., Guo L.Q., Lin J.F. (2022). Ergothioneine Exhibits Longevity-Extension Effect in Drosophila Melanogaster via Regulation of Cholinergic Neurotransmission, Tyrosine Metabolism, and Fatty Acid Oxidation. Food Funct..

[76] Cheah I.K., Ng L.T., Ng L.F., Lam V.Y., Gruber J., Huang C.Y.W., Goh F.Q., Lim K.H.C., Halliwell B. (2019). Inhibition of Amyloid-Induced Toxicity by Ergothioneine in a Transgenic Caenorhabditis Elegans Model. FEBS Lett..

[77] Mirisola M.G., Taormina G., Fabrizio P., Wei M., Hu J., Longo V.D. (2014). Serine- and Threonine/Valine-Dependent Activation of PDK and Tor Orthologs Converge on Sch9 to Promote Aging. PLoS Genet..

[78] Mirzaei H., Suarez J.A., Longo V.D. (2014). Protein and Amino Acid Restriction, Aging and Disease: From Yeast to Humans. Trends Endocrinol. Metab..

[79] Wu Z., Song L., Liu S.Q., Huang D. (2013). Independent and Additive Effects of Glutamic Acid and Methionine on Yeast Longevity. PLoS ONE.

