# Supplementary material for: Anti-Aging and Neuroprotective Properties of Grifola frondosa and Hericium erinaceus Extracts
Source: Nutrients. 2022 Oct 18;14(20):4368. doi: 10.3390/nu14204368 (PMC9611596; doi:10.3390/nu14204368)
Supplement: Supplementary file 1 [file nutrients-14-04368-s001.zip › nutrients-1945939-supplementary.pdf]

Supplementary Materials

Anti-aging and neuroprotective properties of *Grifola frondosa* and *Hericium erinaceus* extracts

Farida Tripodi, Ermelinda Falletta, Manuela Leri, Cristina Angeloni, Daniela Beghelli, Laura Giusti, Riccardo Milanesi, Belém Sampaio-Marques, Paula Ludovico, Lorenzo Goppa, Paola Rossi, Elena Savino, Monica Bucciantini, Paola Coccetti

Supplementary Tables

Table S1. Analytes identified by GC/MS on the basis of match with NIST2014 library and the corresponding target ions used to quantify them.

Analyte		Target ion (m/z)
Crotonic acid		213
Lactic acid		266; 261
1,3-Propandiol or glycolic acid		189
L-Alanine + Sarcosine		260; 158
Glycine		147
Hydracrylic acid		261
L-Valine		288
L-Leucine		274
Isoleucine		200
L-Proline		286
L-Lysine		272; 300
Glyceric acid		391
D-Pyroglutamic acid		272
L-Methionine		292
Adipic acid		317
L-Serine		390

L-Threonine		303
L-Phenylalanine		302; 308; 336
Palmitic acid		313
Aspartic acid		302; 316; 418
Isovanillic acid		339
L-Glutamic acid		432
Azelaic acid		359
Palmitic acid		313
Tetradecanedioic acid		429
Linoelaidic acid		337
Oleic acid		339
Stearic acid		341
Citric acid		459
L-Tyrosine		302
Ergosterol		363; 396

Table S2. Yeast strains used in this study

Strain	Genotype
wt	*BY4742 MATα his3Δ1 leu2Δ0 lys2Δ0 ura3Δ0*
snf1Δ	*BY4742 MATα his3Δ1 leu2Δ0 lys2Δ0 ura3Δ01 snf1::HPH*
atg1Δ	*BY4742 MATα his3Δ1 leu2Δ0 lys2Δ0 ura3Δ01 atg1::KanMX*
ras2Δ	*BY4742 MATα his3Δ1 leu2Δ0 lys2Δ0 ura3Δ01 ras2::KanMX*
tor2Δ	*BY4742 MATα his3Δ1 leu2Δ0 lys2Δ0 ura3Δ01 tor2::KanMX*
wt[empty]	*BY4742 MATα his3Δ1 leu2Δ0 lys2Δ0 ura3Δ0 [pYX242]*
wt[α-syn]	*BY4742 MATα his3Δ1 leu2Δ0 lys2Δ0 ura3Δ0 [pYX242-SNCA]*

Supplementary Figures

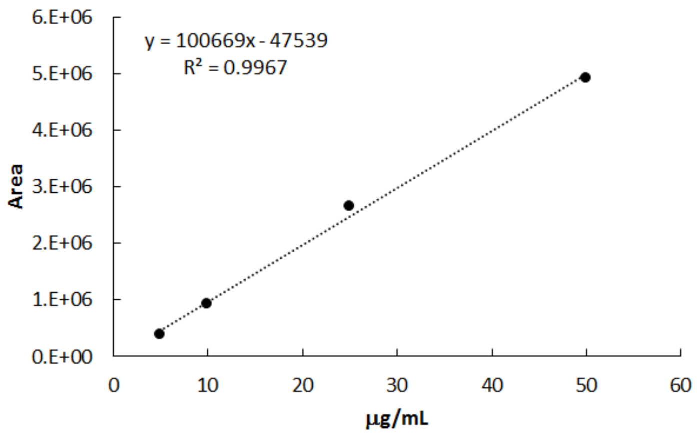

Figure S1. Calibration curve and linear regression curve for ET

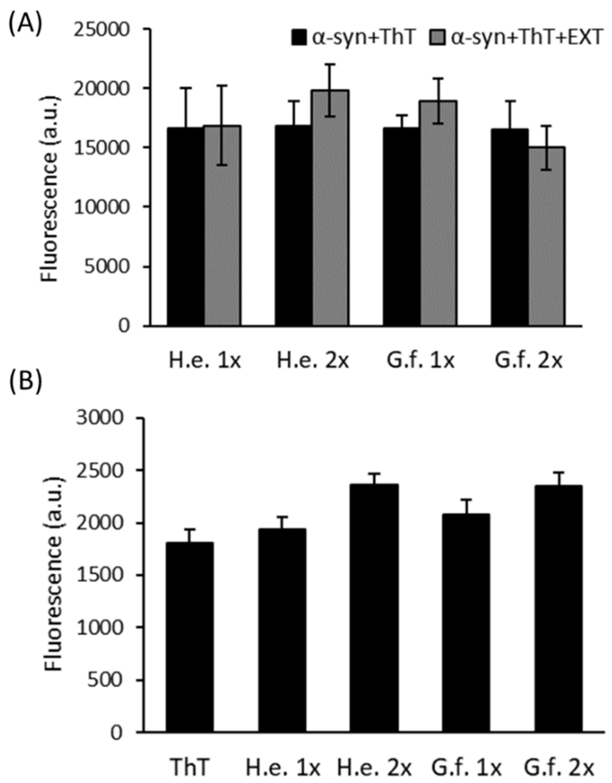

Figure S2. Evaluation of interference of fungal extracts on ThT assay (A) ThT fluorescence intensity recorded in the absence or in the presence of *G. frondosa* or *H. erinaceus* at two α-syn:extract mass ratio (1x and 2x). (B) Addiction of *G. frondosa* or *H. erinaceus* extracts, 1x and 2x, (α-syn+ThT+EXT), on α-syn aggregates pre-incubated with ThT probe (α-syn+ThT) in order to evaluate the possible extracts quenching effects.

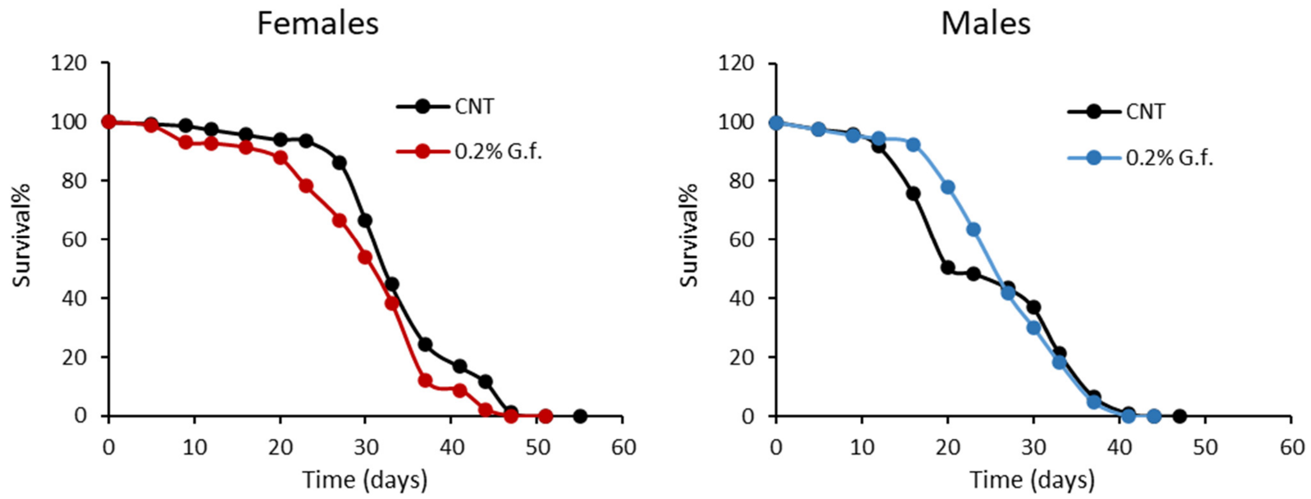

Figure S3. High fungal extract concentrations are toxic for adult flies. Flies were supplemented with 0.2% *G. frondosa* extract lifelong. Data are presented as a percentage of survival of flies as a function of time (in days). The Kaplan-Meier test was used to detect the significant differences among the 2 groups of flies.
